# Comparative vascular responses three months after paclitaxel and everolimus-eluting stent implantation in streptozotocin-induced diabetic porcine coronary arteries

**DOI:** 10.1186/1475-2840-11-75

**Published:** 2012-06-21

**Authors:** Alexander Sheehy, Steven Hsu, Amelie Bouchard, Pablo Lema, Claudine Savard, Louis-Georges Guy, Julie Tai, Igor Polyakov

**Affiliations:** 1 Abbott Vascular, 3200 Lakeside Drive, Santa Clara, CA, 95054, USA; 2 AccelLab Inc, 1635 Lionel-Bertrand, Boisbriand, QC, J7H 1N8, Canada

**Keywords:** Paclitaxel-eluting stent, Everolimus-eluting stent, Restenosis, Vascular response, Percutaneous coronary intervention, Animal model

## Abstract

**Background:**

Diabetes remains a significant risk factor for restenosis/thrombosis following stenting. Although vascular healing responses following drug-eluting stent (DES) treatment have been characterized previously in healthy animals, comparative assessments of different DES in a large animal model with isolated features of diabetes remains limited. We aimed to comparatively assess the vascular response to paclitaxel-eluting (PES) and everolimus-eluting (EES) stents in a porcine coronary model of streptozotocin (STZ)-induced type I diabetes.

**Method:**

Twelve Yucatan swine were induced hyperglycemic with a single STZ dose intravenously to ablate pancreatic β-cells. After two months, each animal received one XIENCE V® (EES) and one Taxus Liberte (PES) stent, respectively, in each coronary artery. After three months, vascular healing was assessed by angiography and histomorphometry. Comparative *in vitro* effects of everolimus and paclitaxel (10^-5^ M–10^-12^ M) after 24 hours on carotid endothelial (EC) and smooth muscle (SMC) cell viability under hyperglycemic (42 mM) conditions were assayed by ELISA. Caspase-3 fluorescent assay was used to quantify caspase-3 activity of EC treated with everolimus or paclitaxel (10^-5^ M, 10^-7^ M) for 24 hours.

**Results:**

After 3 months, EES reduced neointimal area (1.60 ± 0.41 mm, p < 0.001) with trends toward reduced % diameter stenosis (11.2 ± 9.8%, p = 0.12) and angiographic late-loss (0.28 ± 0.30 mm, p = 0.058) compared to PES (neointimal area: 2.74 ± 0.58 mm, % diameter stenosis: 19.3 ± 14.7%, late loss: 0.55 ± 0.53 mm). Histopathology revealed increased inflammation scores (0.54 ± 0.21 *vs.* 0.08 ± 0.05), greater medial necrosis grade (0.52 ± 0.26 *vs.* 0.0 ± 0.0), and persistently elevated fibrin scores (1.60 ± 0.60 *vs.* 0.63 ± 0.41) with PES compared to EES (p < 0.05). *In vitro*, paclitaxel significantly increased (p < 0.05) EC/SMC apoptosis/necrosis at high concentrations (≥10^-7^ M), while everolimus did not affect EC/SMC apoptosis/necrosis within the dose range tested. In ECs, paclitaxel (10^-5^ M) significantly increased caspase-3 activity (p < 0.05) while everolimus had no effect.

**Conclusion:**

After 3 months, both DES exhibited signs of delayed healing in a STZ-induced diabetic swine model. PES exhibited greater neointimal area, increased inflammation, greater medial necrosis, and persistent fibrin compared to EES. Differential effects of everolimus and paclitaxel on vascular cell viability may potentially be a factor in regulating delayed healing observed with PES. Further investigation of molecular mechanisms may aid future development of stent-based therapies in treating coronary artery disease in diabetic patients.

## Background

The prevalence of diabetes is quickly increasing in both developing and developed countries, with the number of diabetic patients expected to increase to 366 million people (4.4% prevalence) globally by 2030 [1]. Coronary artery disease is highly prevalent in diabetic patients and is a major cause of mortality in patients with both type I and type II diabetes [2]. Though the incidence of in-stent restenosis following drug-eluting stent (DES) treatment is greater in diabetic patients compared to non-diabetic patients, several clinical studies have suggested improved prognosis following percutaneous interventions for treatment of coronary and peripheral artery disease in diabetic patients [3,4]. Although the use of DES for treatment of coronary artery disease in patients with diabetes has increased, limited data exists on comparing the vascular responses of different DES in relevant preclinical models of diabetes.

Diabetic patients with coronary artery disease exhibit factors such as hyperglycemia, hyperinsulinemia, hypercholesterolemia, insulin resistance, and increased inflammation [1]. The streptozotocin (STZ)-induced diabetic swine has been described as insulin-controlled hyperglycemia analogous to a type-I diabetic condition. It tends to exhibit increased inflammation but not atherosclerosis [5]. Factors related to insulin resistance and hypercholesterolemia likely differ from a human type II diabetic condition. Other factors important to stenting previously connected to hyperglycemia include increased thrombus and neointima formation [6,7], dysfunctional endothelial cells [8], and impaired wound healing [9] and have previously been demonstrated in this model. Although type I diabetes constitute ~10% of all patients with diabetes, type I diabetics at a young age (≤ 40) may be at high risk for development of severe coronary artery disease [10], and only a few studies have investigated coronary stenting in relevant animal models of type I diabetes.

Preclinical studies have previously characterized vessel healing responses to bare-metal stents (BMS) in STZ-induced type I diabetic animal models and have established delayed healing responses following BMS treatment in STZ animals compared to non-diabetic controls [6,11]. More recent studies have begun to assess the vascular responses of commercially available DES in animal models of type I diabetes. In a STZ rat model comparing first generation sirolimus-eluting stents with paclitaxel-eluting stents (PES), histomorphometric analysis revealed increased thrombus, inflammatory cell infiltration, and neointimal hyperplasia with DES treatment in STZ rats four weeks after aortic stenting compared to non-STZ controls [12]. DES in STZ rats were associated with delayed re-endothelialization, increased fibrin deposition, and changes in extracellular matrix composition compared to DES in non-STZ rats. Although this study was the first study to report comparative effects of DES in a STZ type I diabetic model, use of a large animal model of type I diabetes are advantageous for stent evaluation compared to a small animal model due to development of features with more reliable pathophysiological resemblance to human diabetes and anatomical similarities in vessel size, allowing for assessment of stenting in coronary arteries rather than other vessels with different tissue architecture and flow environments. It has been demonstrated that 6 months after sirolimus-eluting stent implantation in coronary arteries of minipigs with STZ-induced diabetes, the degree of in-stent restenosis, late lumen loss, and neointimal hyperplasia were significantly increased compared to sirolimus-eluting stents in nondiabetic controls [7].

Since previous studies have established delayed healing with both BMS and DES in STZ swine compared to non-STZ controls, we aimed to specifically compare for the first time the *in vivo* vascular response to PES and everolimus-eluting stents (EES) after 90 days in a porcine coronary model of STZ-induced type I diabetes. Paclitaxel is an antineoplastic, lipophilic molecule [13]. Everolimus acts as an antiproliferative and immunosuppressive agent [14]. Vascular responses to PES and EES were assessed by angiography and histomorphometry. To evaluate potential effects of drugs alone on vascular cell viability in support of *in vivo* assessment of DES in hyperglycemic swine, *in vitro* effects of paclitaxel and everolimus on endothelial cell (EC) and smooth muscle cell (SMC) viability were also evaluated under hyperglycemic conditions.

## Methods

### Animals

All experimentation conformed to the Animal Welfare Act and the Guide for Care and Use of Laboratory Animals (NIH Publication 85–23, 1996) and the Canadian Council on Animal Care regulations. All procedures were performed at AccelLab, Inc (Boisbriand, Quebec, Canada), accredited by the Association for Assessment and Accreditation of Laboratory Animal Care and in accordance with the protocol approved by the Institutional Animal Care and Use Committee.

### STZ-induced diabetic porcine model

Twelve Yucatan swine were administered a single dose of STZ (125 mg/kg body weight, Sigma-Aldrich, St. Louis, MO) intravenously to ablate pancreatic β-cells, and blood glucose was monitored daily. Two to three days following STZ injection, daily insulin was used to moderate the increase in fasting glucose from normal (~2 mmol/L) to a target level of 20–23 mmol/L over the course of 4 days using a combination of long-acting (Lantus®) and regulator-acting (Novalin GE Toronto®) insulin and maintained elevated thereafter. Insulin was administered approximately 1 hour after feeding, thus not during fasting prior to procedures.

### Intracoronary stenting

Two months following diabetes induction, stents were implanted and randomized to the left anterior descending (LAD), left circumflex (LCX), or right coronary arteries (RCA) (one stent deployed per artery). A total of 22 stents were deployed: XIENCE V® everolimus-eluting stents (EES, 3.0x12mm, Abbott Vascular, Santa Clara, CA, n = 11) or Taxus Liberte paclitaxel-eluting stents (PES, 3.0x12mm, Boston Scientific, Natick, MA, n = 11). The number of stents was equally divided amongst the three arteries (for EES and PES, 4 in LAD, 3 in LCX, and 4 in RCA), with placement of stents within coronary arteries similar for all groups. However, stents were not implanted in the exact same anatomical locations within each coronary artery (*e.g.* after first diagonal) but were implanted based upon similar artery size. There were only 11 arteries evaluated per treatment group, as arteries less than 2.6 mm in diameter were excluded to ensure a stent-to-artery ratio of 1.1:1 and full apposition of all stents was achieved angiographically in all coronaries devoid of major side branches.

### Procedure

Animals were administered oral acetylsalicylic acid (325 mg) and clopidogrel (300 mg initial dose and 75 mg subsequently) beginning three days prior to stent implantation and continuing daily until sacrifice. Animals were tranquilized with ketamine (0.04 mg/kg), azaperone (4.0 mg/kg), and atropine (25 mg/kg) intramuscularly. Anesthesia was achieved with propofol (1.66 mg/kg IV), and maintained with isofluorane (1-3%) throughout the procedure. A vascular access sheath was placed in the femoral artery percutaneously. Before catheterization, heparin (400U/kg) was injected to maintain an activated clotting time >300 s. At three months post-stenting, follow-up angiography was performed. Three months was chosen as the earliest time point at which differences in arterial healing response between DES may emerge [15] and because 90 days in swine has been suggested to correspond to >1 year in humans [16]. Animals were euthanized under general anesthesia. Hearts were excised and pressure-perfused with 0.9% saline followed by pressure-perfusion fixation in 10% neutral buffered formalin.

### Animal health

Blood was collected prior to diabetes initiation, at stent implant, and prior to follow-up angiography for serum biochemistry and hematology (Marshfield Laboratories, Marshfield, WI). Upon termination, gross necropsy was performed and samples of the kidney, lung, liver, and pancreas were stained with hematoxylin and eosin (H&E) and evaluated by a trained pathologist. Observations of the kidney, liver, lung, and pancreas were noted at necropsy and from histopathological assessment of H&E stained tissue sections using light microscopy. Pathophysiological observations in each tissue were described and when applicable received a subjective score (0 = absent or not present, 1 = mild, 2 = moderate, 3 = marked) following evaluation of H&E tissue staining from the blinded study pathologist.

### Histological assessment of stented coronary artery tissue in STZ-induced diabetic porcine

The fixed stented arterial segments were dehydrated in a graded series of ethanol and embedded in methyl methacrylate resin. For each stent, three sections (proximal, middle, and distal) were cut on a rotary microtome (~5 μm thickness) and stained with H&E and elastic Van Gieson stains. Proximal and distal sections to the stented region were stained with H&E.

The cross-sectional areas (internal elastic lamina (IEL) and lumen) of each section were measured using digital morphometry software (Image Pro, Media Cybernetics, Bethesda, MD). Neointimal area was measured on elastin stained sections. The IEL was traced as was the lumen, and the neointimal area was calculated as the IEL area minus lumen area. Histopathology scoring of pre-specified criteria for: injury [17], inflammation, fibrin, and neointimal immaturity was performed on a 0–3 scale by a trained pathologist independent of the study sponsor through blind review and peer-reviewed by a second pathologist. For inflammation and fibrin, a score of 0 indicated absent or rare occurrence around a strut, while a score of 3 indicated most severe around a strut as well as abundant between struts in surrounding tissue. Neointimal immaturity scoring was based upon the proportion of area with immature healing and organization in response to stenting. Less mature areas were scored as 3 and identified as containing few to no myofibroblasts, a high proportion of mucinous matrix, edema, or fibrin, and undifferentiated mesenchymal cells or inflammatory cells.

Histopathological samples were evaluated semi-quantitatively for strut/IEL gap, peri-strut hemorrhage, peri-strut plasma, marginated/subendothelial leukocytes, neointimal neovascularization, medial necrosis, adventitial inflammation, and adventitial fibrosis by a blinded study pathologist independent of the study sponsor and peer-reviewed by a second pathologist *via* hematoxylin and eosin (H&E)/Van Gieson stained stented arterial segments using light microscopy. Observations were scored subjectively (0 = absent or not present, 1 = mild, 2 = moderate, 3 = marked) when applicable, or the percentage of struts affected was enumerated. The incidence of each observation, or when applicable, the group mean score, was tabulated and reported by stent group.

### *In vitro* effects of paclitaxel and everolimus on SMC and EC apoptosis/necrosis

EC and SMC were harvested from carotid artery of Yucatan swine with an enzymatic digestion procedure described previously [18] and cultured in Dulbecco’s Modified Eagle Medium (DMEM) media (Lonza, Walkersville, MD) supplemented with 10% fetal bovine serum (FBS) and 1% penicillin-streptomycin (Invitrogen, Carlsbad, CA). Cells were cultured in a 37°C and 5% CO_2_ environment, and all experiments were performed using cells at passage 6 or lower. EC were immunohistologically characterized by positive PECAM-1/CD31 (Abcam, Cambridge, MA) staining, while SMC were characterized by positive SM actin, SM myosin, and calponin (all Dako, Carpinteria, CA) staining (data not shown).

To assess the effects of everolimus and paclitaxel on EC and SMC viability, total cell necrosis and induction of apoptosis were evaluated using a Cell Death Detection ELISA^PLUS^ kit (Roche Applied Science, Mannheim, Germany), with an endpoint of absorbance (A_405nm_ – A_490nm_) read on a M5 Spectramax plate reader (Molecular Devices Corporation, Sunnyvale, CA) according to manufacturer’s specifications. On Day 0, EC or SMC were seeded in 24-well plates (Costar, Lowell, MA) and allowed to grow to 80% confluence under high glucose conditions (42 mM, Sigma-Aldrich) similar to hyperglycemic levels used in previous studies [19,20]. Cells were then treated with dimethyl sulfoxide (DMSO, Sigma-Aldrich) vehicle control, everolimus (Novartis, Basel, Switzerland), or paclitaxel (Sigma-Aldrich) over a range of concentrations (10^-5^ M–10^-12^ M), or growth media alone (control) for 24 hours. Following treatment, cell media supernatants were collected, cultures were washed with PBS, and cells were treated with lysis buffer supplied in ELISA kit. ELISA detection of histone-associated nucleosome concentration in cell media supernatants was used to determine overall necrosis, while concentration in cell lysates was used to determine relative induction of apoptosis, all according to manufacturer’s instructions. A caspase-3 fluorescent assay kit (Clontech Laboratories, Mountain View, CA) was used to quantify caspase-3 activity of EC cultured under high glucose conditions (42 mM) and treated with everolimus or paclitaxel (10^-5^ M, 10^-7^ M) for 24 hours. Total protein content of cell lysates was determined using Bradford DC Protein assay (Bio-Rad Laboratories, Hercules, CA), with an endpoint of absorbance (A_750nm_) read on an M5 Spectramax plate reader. Apoptosis, necrosis, and caspase-3 activity data for each sample were normalized to respective total protein content and expressed as fold change relative to control samples.

### Statistical analysis

All data are presented as mean ± standard deviation unless otherwise specified. All data was analyzed according to criteria pre-specified in the study protocol. For all data, equal variance test and normality tests were performed prior to other mean comparison testing. For normal data, a one way analysis of variance (ANOVA) was used with Holm’s post-hoc test to compare differences between groups. For non-parametric data, a Wilcoxon rank-sum test was used to compare between two samples. A p-value of p < 0.05 was considered statistically significant for evaluating differences between treatment groups.

## Results

### Animal health

Following STZ injection, animals were hypoglycemic below the threshold of detection (0.6 mmol/L). Within a few days, animals became hyperglycemic and were maintained hyperglycemic (10-23 mmol/L) throughout the remaining 5 months. Animals had an average weight loss of 11.1 ± 5.0 kg during the initial two months of diabetes and stabilized thereafter (average gain 2.7 ± 6.4 kg). Animals exhibited altered blood serum due to diabetes as summarized in Table 1. No other abnormal blood work was observed. Based upon histopathologic evaluation following euthanasia, 9/12 swine had evidence of mild to marked tubulointerstitial degeneration and 2/12 had chronic mild to moderate glomerulosclerosis. Evidence of pancreatic β-cell atrophy was observed in all animals.

**Table 1:** Assessment of blood serum chemistry

**Serum**	**Baseline**	**2 months (pre-stent)**	**5 months (terminal)**
Bilirubin	0.31 ± 0.28 μM	4.40 ± 2.43 μM *	1.42 ± 1.23 μM *
Blood Urea Nitrogen	5.4 ± 1.1 mM	7.0 ± 1.0 mM *	8.4 ± 2.7 mM *
Serum Creatinine	72 ± 15 μM	105 ± 45 μM *	91 ± 30 μM *

Swine were healthy through the remainder of the study with only altered blood work related to diabetes: n = 12. * p < 0.05 *vs.* baseline.

### QCA

All animals survived stent implantation and follow up procedures without complication. There were no differences between groups in pre-stent reference vessel diameter or post-stent minimal lumen diameter (MLD) as measured by QCA. Table 2 summarizes the QCA results for all groups. There was a trend towards increased late loss with PES (p = 0.058) in comparison to EES.

**Table 2:** QCA assessment of coronary arteries

**In stent**	**Xience V**	**Taxus**	**p-value**
Pre-Stent Mean Lumen Diameter	2.68 ± 0.19	2.79 ± 0.19	-
Balloon to artery ratio	1.16 ± 0.03*	1.10 ± 0.03	0.002
MLD, mm	2.40 ± 0.30	2.26 ± 0.50	0.25
Diameter stenosis, %	11.2 ± 9.8	19.3 ± 14.7	0.12
Binary restenosis, %	0	8.3	N/A
Late Loss, mm	0.28 ± 0.30	0.55 ± 0.53	0.058

MLD – Minimal lumen diameter. n = 11 for Xience V, n = 11 for Taxus.

* p < 0.05 *vs.* Taxus.

### Histomorphometry

Histomorphometry confirmed trends observed by QCA. PES displayed increased neointimal area (2.74 ± 0.58 mm, n = 11) in comparison to EES (1.60 ± 0.41 mm, n = 11) (p < 0.001). No significant differences in percent area stenosis were observed between PES (37.9 ± 10.4%) and EES (26.3 ± 8.0%) treatment groups. Complete histomorphometric findings are summarized in Table 3.

**Table 3:** Histomorphometry assessment of coronary arteries 3 months post-stenting in STZ-induced diabetic swine

	**Xience V**	**Taxus**	**p-value**
EEL Area (mm^2^)	7.33 ± 0.92 *	8.44 ± 0.88	0.022
IEL Area (mm^2^)	6.22 ± 0.77 *	7.35 ± 0.74	0.005
Medial Area (mm^2^)	1.12 ± 0.23	1.09 ± 0.22	-
Intimal Area (mm^2^)	1.60 ± 0.41 *	2.74 ± 0.58	<0.001
Luminal Area (mm^2^)	4.62 ± 0.94	4.61 ± 1.06	-
Area Stenosis (%)	26.3 ± 8.0	37.9 ± 10.4	-

EEL – External elastic lamina. IEL – Internal elastic lamina. n = 11 for Xience V, n = 11 for Taxus. * p < 0.05 *vs.* Taxus.

### Histopathology

**Figure 1:**
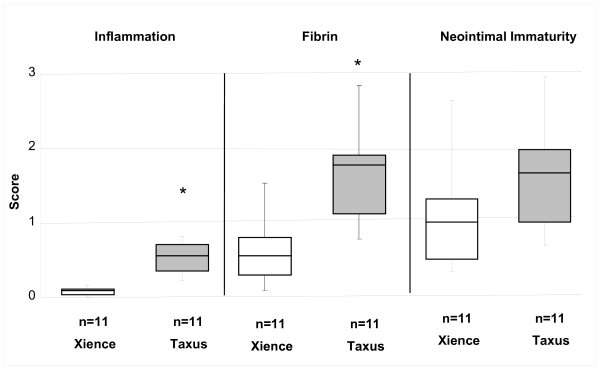
** Histological assessment of coronary artery tissue stented with PES and EES in STZ-induced type I diabetic swine.** Histopathological scoring (mean ± standard deviation, n = 11) revealed delayed neointimal healing in response to PES after 90 days. Both A) inflammation and B) fibrin were significantly increased in response to PES (Taxus) (p < 0.05) compared to EES (Xience V). C) No significant differences in neointimal immaturity were found between PES and EES. ^*^ p < 0.05

**Figure 2:**
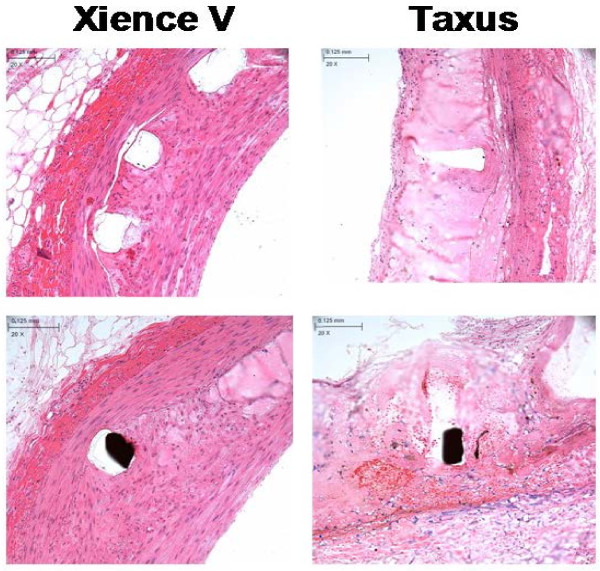
** Histopathology of coronary artery tissue stented with PES and EES in STZ-induced type I diabetic swine.** Representative histopathological microscopy images (20x) of coronary arteries stented with EES (Xience V) and PES (Taxus) and stained with H&E after 90 day implantation

Characteristics observed in both DES suggested delayed healing in these STZ-induced type I diabetic animals in comparison to our previous experience in healthy swine as well as that of others [21]. There were no significant differences in injury scores across the two devices (EES 0.18 ± 0.16, PES 0.09 ± 0.13, p = 0.279). Inflammation was moderately increased in response to PES (0.54 ± 0.21, n = 11) compared to EES (0.08 ± 0.05, p < 0.05 *vs.* PES, n = 11) (Figure 1A). The predominant inflammatory infiltrate was macrophages with occasional multinucleated giant cells. Fibrin scores were also significantly increased (p < 0.05) with PES (1.60 ± 0.60) when compared to EES (0.63 ± 0.41) (Figure 1B). Both DES exhibited neointimal immaturity (Figure 1C), with no significant differences observed between EES (1.06 ± 0.67) and PES (1.64 ± 0.69). Immature neointima was characterized by marginated subendothelial leukocytes, amorphous fibrin, and peri-strut hemorrhage and hemosiderin deposits. Table 4 characterizes the composition and distribution of histopathologic findings. In PES, fibrin created a gap between the IEL and the strut in 7 of 11 vessels in approximately 1/3 of the struts. Medial necrosis was greater in PES struts in comparison to EES (Table 4, Figure 2). In EES, accumulation of homogenous pale eosinophilic material was present around the struts (11/11 vessels) while infrequent in PES (3/11 vessels). This fluid rarely contained cells and was interpreted as extra-cellular fluid of blood plasma origin.

**Table 4:** Histopathology composition and distribution of findings

		**Xience V**	**Taxus**
Strut/IEL gap	Incidence	1/11	7/11
	Percentage	5.6	36.9 ± 32.9
Peri-strut hemorrhage	Incidence	------	2/11
	Percentage	------	11.3 ± 10.7
Peri-strut plasma	Incidence	11/11	3/11
	Grade	1.35 ± 20.6	0.78 ± 0.39
Marginated/subendothelial leukocytes	Incidence	4/11	10/11
	Grade	0.46 ± 0.16	1.00 ± 0.52
Neointimal neovascularization	Incidence	------	5/11
	Grade	------	0.80 ± 0.18
Medial necrosis	Incidence	------	7/11
	Grade	------	0.52 ± 0.26
Adventitial inflammation	Incidence	------	1/11
	Grade	------	0.33
Adventitial fibrosis	Incidence	------	------
	Grade	------	------

Incidence results are presented as # of stents with findings/total number of stents.

Mean grade or percentage results are presented as mean grade or percentage of affected struts per group ± standard deviation.

n = 11 for Xience V, n = 11 for Taxus.

### *In vitro* effects of everolimus and paclitaxel on EC and SMC apoptosis/necrosis

**Figure 3:**
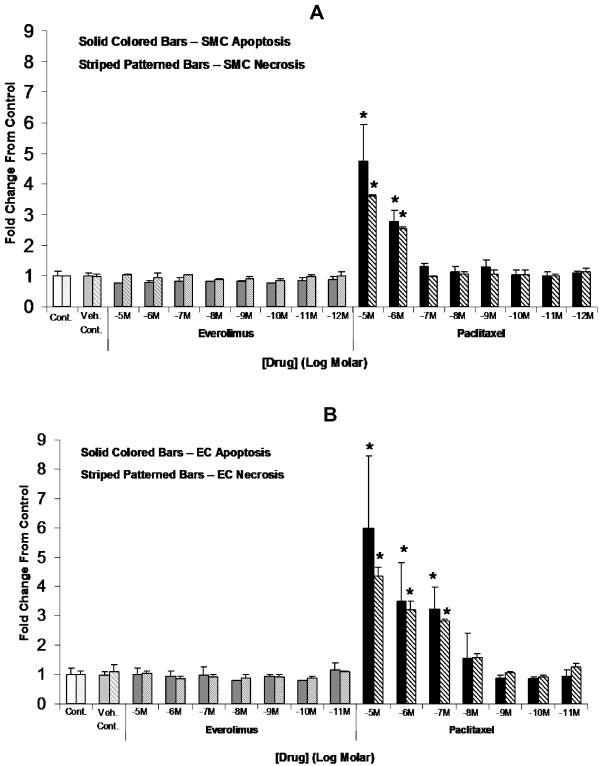
** Effects of paclitaxel and everolimus on SMC and EC apoptosis and necrosis.** SMC or EC were cultured *in vitro* under hyperglycemic conditions (42 mM) in 24-well plates and allowed to grow to 80% confluence. Cells were then treated with growth media alone (control), DMSO vehicle control, or everolimus or paclitaxel over a range of concentrations (10^-5^ M–10^-12^ M) for 24 hours. A Cell Death Detection ELISA^PLUS^ kit was then used to detect histone-associated nucleosome concentration in cell media supernatants to determine overall necrosis, while concentration in cell lysates was used to determine relative induction of apoptosis. Data expressed as fold change relative to control (mean ± standard deviation, n = 3). **A**) Paclitaxel significantly increased SMC apoptosis and necrosis in a dose-dependent manner at concentrations of 1 μM and greater. **B**) Paclitaxel significantly increased EC apoptosis and necrosis in a dose-dependent manner at concentrations of 0.1 μM and greater. Everolimus did not increase SMC or EC apoptosis or necrosis at any dose tested. ^*^ p < 0.05 *vs.* control

SMC were cultured in high glucose conditions to ascertain effects of everolimus and paclitaxel on apoptosis and secondary necrosis. After 24 hours, quantitative measurement of apoptosis from cellular lysates revealed everolimus did not significantly increase SMC apoptosis at any dose tested (10^-5^ M-10^-12^ M, n = 3) compared to controls (Figure 3A). Paclitaxel significantly increased apoptosis of SMC from STZ swine at both 10^-5^ M (4.75 ± 1.17-fold, p < 0.05) and 10^-6^ M (2.77 ± 0.38-fold, p < 0.05) compared to controls and everolimus-treated groups. Similar results were obtained when cell media supernatants were analyzed to assess drug effects on SMC necrosis (Figure 3A).

**Figure 4:**
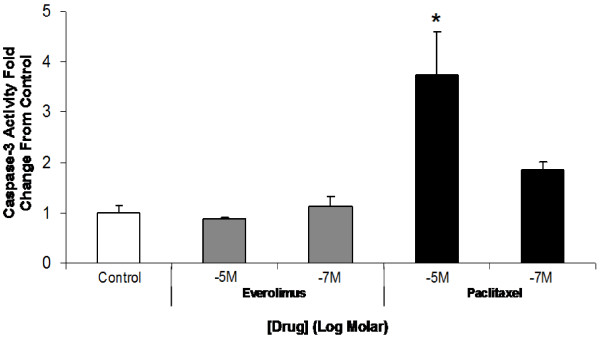
** Effects of paclitaxel and everolimus on caspase-3 activity in EC.** EC were cultured *in vitro* under hyperglycemic conditions (42 mM) and allowed to grow to 80% confluence. Cells were then treated with growth media alone (control), or everolimus or paclitaxel (10^-5^ M, 10^-7^ M) for 24 hours. A caspase-3 fluorescent assay kit was used to quantify caspase-3 activity. Data expressed as fold change relative to control (mean ± standard deviation, n = 3). Paclitaxel increased EC caspase-3 activity in a dose-dependent manner while everolimus had no effect. ^*^ p < 0.05 *vs.* control

Experiments to examine the effects of everolimus and paclitaxel on apoptosis and necrosis of EC under high glucose conditions revealed similar trends in drug effects as those observed in SMC (Figure 3B). Compared to SMC, EC appeared to be more sensitive to paclitaxel treatment. At 10^-7^ M paclitaxel, elevated apoptosis/necrosis was observed in EC but not in SMC, and the extent of apoptosis/necrosis was greater in EC compared to SMC in response to paclitaxel at higher concentrations (Figure 3A and 3B). Paclitaxel, but not everolimus, at 10^-5^ M (n = 3, p < 0.05) significantly upregulated caspase-3 in ECs, suggesting that paclitaxel-induced EC apoptosis was caspase-3 dependent (Figure 4).

## Discussion

In the present study, we compared the coronary arterial healing to paclitaxel- and everolimus-eluting stents in a porcine model of STZ-induced type I diabetes in non-overlapping segments 90 days following stent implantation and investigated *in vitro* drug effects on EC and SMC viability. The main findings of our study were: 1) in the presence of chronic hyperglycemia, in comparison to everolimus-eluting stents, paclitaxel-eluting stents exhibited signs of delayed healing, notably greater neointimal area, increased inflammation, greater medial necrosis, and persistent fibrin, after 3 months and 2) paclitaxel, unlike everolimus, significantly increased EC and SMC apoptosis/necrosis *in vitro* at high concentrations (≥10^-7^ M) under hyperglycemic culture conditions.

### Animal health

Overall, animals remained in good health throughout the study with systemic abnormalities that were attributable to type I diabetes induction including alterations to blood proteins and changes in the kidney. The observed histopathological changes in the kidney were indicative of early stages of diabetic nephropathy [22].

### *In vivo* histopathology

Histopathologically, percent stenosis was found to be numerically greatest but not significantly different in PES in comparison to EES. One of the first histopathologic evaluations of PES deployed in rabbit iliac arteries demonstrated a reduction in mean neointimal thickness at 28 days but not at 90 days [23]. Similarly, in healthy swine, PES have demonstrated decreased neointima at 30 days but not 90 days [21]. Pathology of arteries stented with PES has characterized the neointima as containing persistent intimal fibrin deposition, intraintimal hemorrhage, and increased inflammation [23]. Increased fibrin deposition associated with PES has also been reported following stenting in human coronary arteries [24]. Interestingly, in a reported dose dependent study [23], the histologic findings of delayed healing were not present in the lower dose PES group, suggesting that drug effect was a primary culprit in the pathology herein. Other researchers have similarly reported on the significant delayed healing in stents eluting paclitaxel in healthy rabbits [25] and swine [26]. Most recently, in a diabetic/hypercholesterolemic porcine model, the coronary responses to BMS and PES implantation were reported to display increased inflammation with SMC loss and persistent fibrin out to 90 days [27]. Compared to BMS, PES also exhibited increased neointimal area and delayed reendothelialization. Similar to these previous studies, in this model of diabetes without hypercholesterolemia, PES exhibited increased neointima and delayed healing in comparison to an everolimus-eluting stent.

The findings following stenting with an everolimus-eluting stent support previous published work with this device as well. Low inflammation and endothelialization have been reported following everolimus-eluting stent implantation in healthy rabbits [28] and swine [29,30] as well as in atherosclerotic rabbits [31]. Other investigators have described the vessel healing response to everolimus-eluting stents in overlap configurations as well [32]. In particular, the presence of fibrin following stenting with everolimus-eluting stents was not different than bare metal stents 90 days following stenting in a porcine model in single and overlap configurations. The data in the present study suggest that the EES continues to heal in spite of chronic hyperglycemia out to 90 days in a swine model. Fibrin was observed to be minimal at 90 days with inflammation almost completely resolved.

In addition to previous findings reported with these stents, we report here an additional description of increased neovascularization in PES, which is a common finding following thrombus/fibrin accumulation and accompanied inflammation and has been suggested to be directly related to the early phases of thrombus healing [33]. The EES vessels occurred without significant neovascularization, likely the result of less fibrin reorganization earlier in the healing process. The inflammatory response to both PES and EES primarily characterized as para-strut macrophages with occasional giant cells and few lymphocytes has been characterized previously as the predominant inflammatory response to stenting [34,35] in humans. The magnitude rather than the composition of inflammation represents the differential inflammatory response to these two devices. Peri-strut eosinophilic material interpreted as plasma due to its morphology, staining, and paucity of cells was found most frequently in EES and has seldom been reported pathologically following stenting. This finding may be an indication of endothelial cell leakage from an immature endothelial cell layer. Additional research is warranted to understand the temporal differences in healing following stenting with everolimus-eluting stents in a diabetic swine model as well as the effects of hypercholesterolemia in comparison to a healthy model.

### Differential effects of everolimus and paclitaxel on EC and SMC apoptosis/necrosis

Since our *in vivo* comparison of vascular responses to PES and EES was solely limited to STZ swine, we solely characterized potential differences of drug alone on vascular EC and SMC viability under hyperglycemic culture conditions. *In vitro*, our results demonstrate differential effects of everolimus and paclitaxel on the viability of ECs and SMCs cultured under high glucose conditions. Everolimus did not affect apoptosis or necrosis of EC or SMC, while paclitaxel significantly increased apoptosis and necrosis of both EC and SMC at high concentrations (≥10^-7^ M). Additionally, our data demonstrated that paclitaxel-induced apoptosis in ECs under high glucose conditions may occur through caspase-3 activation. Since the concentrations of paclitaxel that were observed to impact EC and SMC viability are potentially achievable through local stent-based delivery [36,37], the differential effects of these drugs on EC and SMC viability may be a contributing factor to the delayed healing of PES we observed in STZ-induced diabetic swine compared to EES. Previously, paclitaxel has been found to regulate the cytotoxicity of vascular cells under non-hyperglycemic culture conditions [38]. Increased SMC apoptosis/necrosis can increase cellular debris, induce inflammation, and increase risk of thrombosis [39]. Dose-dependent effects of paclitaxel have been shown to increase fibrin deposition and medial necrosis following PES treatment in rabbit and porcine models [23,40], which mirror our histopathological observations with PES exhibiting both greater medial necrosis and fibrin score compared to EES. Investigators have linked paclitaxel-induced apoptosis [41] to p-Akt reduction in SMC and speculated that Akt may play a vasoprotective role in the histopathologic response to stenting in diabetes [11]. Hypoactivation of Akt has been previously linked with increased cell proliferation, increased inflammation, and decreased cell viability [5,11,42]. In ECs, the decrease in cell viability due to paclitaxel treatment was correlated with a decrease in caspase-3 activity, which potentially may be attributed to the Akt pathway through Gas6-Axl interactions, as has been shown previously in human umbilical vein EC [43]. Further cell culture viability studies are needed to elucidate if similar links between Akt and downstream caspase-3 activation occur in response to these drugs under hyperglycemic culture conditions.

Despite our *in vitro* observations on the effects of drugs alone on vascular cell viability and the correlation with *in vivo* observations, it is important to recognize that DES systems are complex and consist of multiple components. Therefore, we cannot solely attribute the differences in vascular responses observed *in vivo* between PES and EES to drug effects alone, as the exact mechanisms for the apparent differences observed are most likely multifactorial. Though our *in vitro* data indicate that drug may be a contributing factor in causing the histologic differences observed between PES and EES, other contributing factors could include differences in coating, drug release kinetics, drug dose levels, stent configuration impacting blood flow, strut thickness, degree of hyperglycemia, and anatomical lesion location. For instance, based upon previous studies, polymer-free DES exhibited decreased fibrin deposition and inflammation compared with polymer-based DES coatings [44]. The differences in inflammation observed with EES compared with PES in our study may be attributable to differences in coating. Different stent configurations and strut thicknesses are capable of impacting EC growth and subsequent endothelial coverage by impacting blood flow and wall shear stress [28,45]. Further studies are needed to better characterize the effects of these individual factors in regulating *in vivo* vascular response to DES.

### Study limitations

This study has several potential limitations. By using STZ to induce diabetes in swine, we created a large animal model of type I diabetes with ablation of pancreatic β-cells that shares clinical similarities of chronic hyperglycemia. However, type II diabetes represents a complex mixture of additional systemic (obesity, hyperlipidemia, hyperinsulinemia) components. Though no preclinical model can completely mimic the complexity of human disease, this study isolates the effects of hyperglycemia in comparing the arterial response to different DES in a large animal model. Since significant prior data exists in evaluating DES response in healthy animal models, the exclusion of healthy control animals in this study should not invalidate the results of this study, as the primary goal of this study was to compare for the first time the vascular responses of two commercially available DES with differing drug mechanisms of action in a large diseased animal model of diabetes. It is important to acknowledge that although results from preclinical animal models do not always correlate with clinical results due to differences in vessel disease development in humans, this study demonstrated the ability of using a STZ swine model to characterize differential vascular responses between PES and EES. It should be noted that clinical trials comparing EES to PES in diabetes have shown no reduction in target lesion failure and target lesion revascularization [46,47]. An area of future study will be to evaluate delayed healing following stenting in other complex models to better understand the contribution of each factor to stent pathology. Lastly, though we compared effects of paclitaxel and everolimus *in vitro* to evaluate differential effects of drug alone on vascular cell viability, it would be interesting to assess if similar responses to drugs would be observed using arterial tissue from a diabetic animal model.

## Conclusions

The porcine model of STZ-induced type I diabetes allows insight into comparing the differential vascular response to PES and EES in a chronic hyperglycemic state. After 3 months, PES exhibited greater neointimal area, increased inflammation, greater medial necrosis, and persistent fibrin compared to EES. The differential *in vitro* effects of paclitaxel in reducing SMC/EC viability under high glucose conditions compared to everolimus may contribute to the delayed healing observed with PES in a STZ swine model. Further studies are needed to assess the intracellular signaling mechanisms involved in regulating differential vascular responses between DES in diabetic animal models, which would aid future development and evaluation of stent-based therapies in treating coronary artery disease in diabetic patients.

## Competing interests

A. Sheehy, S. Hsu, J. Tai, and I. Polyakov are employees of Abbott Vascular. A. Bouchard, P. Lema, C. Savard, and L.G. Guy are employees of AccelLab, Inc. AccelLab, Inc. receives sponsored study research support from Abbott Vascular, Biotronik, Boston Scientific, Medtronic, Baxter, Atrium Medical, Arsenal Medical, Cardio3 Biosciences, Elixir Medical, Cordis, and Avantec.

## Authors’ contributions

AS participated in the overall design, data interpretation, writing and presentation of this work. SH participated in design of *in vitro* cell viability experiments, data interpretation, performed statistical analysis, drafted portions of the manuscript, and critically revised the manuscript before final approval. AB contributed to the *in vivo* data collection and interpretation. PL was the attending veterinarian, contributed to the model design, and overall conduct of the study. CS contributed to the pathological evaluation and interpretation. LG contributed to the overall *in vivo* study design and data interpretation. JT participated in design of *in vitro* cell viability experiments, data interpretation, and critically revised the manuscript before final approval. IP contributed to the *in vivo* study design, data interpretation, and pathology peer review. All authors read and approved the final manuscript.
